# Protection of Prior SARS-CoV-2 Infection Against Different Variants, Including Omicron Descendants, in a Country with High Viral Transmission

**DOI:** 10.20411/pai.v10i2.760

**Published:** 2025-05-15

**Authors:** Stefan Escobar-Agreda, Roger V. Araujo-Castillo, Luis Pampa-Espinoza, Javier Silva-Valencia, Lely Solari

**Affiliations:** 1 Centro Nacional de Salud Publica, Instituto Nacional de Salud, Lima, Peru

**Keywords:** COVID-19, Reinfection, SARS-CoV-2 variants, Omicron SARS-CoV-2 variant, Peru

## Abstract

**Background:**

Prior infection with SARS-CoV-2 has been reported to confer protection against reinfections. Because Peru has been affected by several variants of this virus, it is an ideal location to better explore this phenomenon. In this study, we aim to evaluate protection of prior SARS-CoV-2 infection against reinfection by variants during the COVID-19 pandemic in Peru.

**Methods:**

A nested case-control study was carried out, using national data from Peru between 2021 and 2023. Five study periods were defined, delimited by the predominance of the main SARS-CoV-2 variants circulating during the pandemic. Cases were paired with controls in a 1 to 4 rate by sex, age, region, being a health worker, and the week of infection. Protection was calculated using conditional logistic regression to estimate odds ratios (OR) with 95% confidence intervals (95% CI) expressed as (1-OR) x100.

**Results:**

Protection from prior infection against SARS-CoV-2 reinfection was 86.3% (95% CI, 81.8 to 89.7) for Lambda, 73.0% (95% CI, 62.9 to 80.3) for Gamma, 84.7% (95% CI, 82.1 to 86.9) for Delta, 34.9% (95% CI, 25.5 to 43.1) for Omicron BA.1, 67.0% (95% CI, 58.7 to 73.6) for Omicron BA.2.12.1, 49.1% (95% CI, 40.5 to 56.5) for Omicron BA.4, 44.8% (95% CI, 39.9 to 49.3) for Omicron BA.5, 29.4% (95% CI, 18.2 to 39.1) for Omicron BQ, and 8.6% (95% CI, -0.5 to 16.9) for Omicron XBB.

**Conclusions:**

Prior infection provides significant protection against SARS-CoV-2 reinfection episodes, although this varies widely among the different Omicron sublineages.

## INTRODUCTION

SARS-CoV-2, the virus that causes COVID-19, has triggered multiple waves of infections worldwide [1], resulting in a substantial socioeconomic and health burden [2, 3]. One critical factor that has facilitated the virus's continued spread is the emergence of new variants [4] that possess advantages over the original lineage, including the ability to cause reinfections despite prior infection-induced immunity [5]. While some studies have explored the extent of protection offered by prior infection against SARS-CoV-2 variants such as Alpha, Beta, Delta, and some Omicron sublineages [6–8], there is limited evidence on the protection against other variants such as Gamma (P.1), Lambda (C.37), and the latest Omicron sublineages BQ and XBB, despite causing significant outbreaks and an increased prevalence of reinfections in some countries, including Peru [9, 10].

In response to this challenge, the Peruvian National Institute of Health (INS in Spanish) has implemented a genomic surveillance system since 2021 to monitor the spread of different SARS-CoV-2 variants across the country. Based on this information, our study seeks to evaluate the protection offered by prior infection against SARS-CoV-2, with a specific focus on the circulating variants during the COVID-19 pandemic in Peru between 2021 and 2023. In addition, we aim to investigate whether this protection varies according to the duration of time since the prior infection.

## METHODS

### Study Design

We conducted an observational study using a nested case-control design based on the test-negative approach. We used data from the Peruvian national registers of antigen tests (SISCOVID) and molecular tests (NETLAB 2.0) for SARS-CoV-2 diagnosis, as well as the national health worker registry (INFORHUS) and the national COVID-19 vaccination registry from the Peruvian Ministry of Health (MoH). The process of integrating these databases is outlined in Figure S4 in Supplementary Appendix.

We defined 5 study periods from May 2, 2021 and February 11, 2023 based on the predominance of circulating SARS-CoV-2 variants in Peru (See S1.2 in Supplementary Appendix). The first period to evaluate reinfection by Lambda and Gamma variants was from the 18^th^ week (May 2) to the 39^th^ week (October 2) of 2021. The second period to evaluate reinfection by Delta variant was from the 40^th^ week (October 3) to the 52^nd^ week (December 25) of 2021. The third period to evaluate the reinfection by the Omicron BA.1 variant was from the 52^nd^ week (December 26) of 2021 to the 13^th^ week (April 23) of 2022. The fourth period to evaluate the reinfection by the Omicron BA.2.12.1, BA.4, and BA.5 variants was from the 14^th^ week (April 24) to the 41^st^ week (October 15) of 2022. Finally, the fifth study period to evaluate the reinfection by the Omicron BQ and XBB variants was from the 42^nd^ week (October 16) to the sixth week (February 11) of 2023.

### Study Population and Variables

We included symptomatic participants aged 0 to 100 years living in Peru who underwent a molecular test (Rt-PCR) during each study period. Symptom status was recorded at the time of testing in the SISCOVID database through a standardized variable indicating whether the individual reported any COVID-19-related symptoms (eg, fever, cough, shortness of breath). Based on the first molecular test result for each study period, cases were defined as individuals who tested positive for the SARS-CoV-2 variant under study, and controls were those with a negative result. Cases were matched with controls in a 1:4 ratio according to sex, age (in 10-year intervals), region of origin, and health worker status. Information about laboratory methods used to identify cases (PCR) and variants (genome sequencing) are detailed in Section S3 of the Supplementary Appendix.

The exposure of interest was a history of prior infection, defined as having a positive antigen or molecular test at least 90 days before their first molecular test in the corresponding study period. Due to the extensive use of serologic testing in early 2020, we also included individuals with a positive serologic test from that period as prior infections. Individuals with infections within 90 days prior to the test were excluded from the study. Detailed information on case/control selection and matching by study group is presented in Figure S5-S9 (Supplementary Appendix).

To describe the dynamics of infection and reinfection cases in Peru, we calculated the frequency of total infections (positive PCR, Antigen or Serologic test), possible reinfections (2 positive PCR or Antigen tests ≥90 days apart), and probable reinfections (2 positive PCR tests ≥90 days apart)e using established definitions [11].

Beyond sex and age, we also included other individual characteristics such as region of origin, health worker status, and vaccination status. All variables included in the study, along with their coding, are detailed in Table S2 in the Supplementary Appendix

### Analysis and Ethical Considerations

A descriptive analysis was performed to characterize the population across the 5 study periods. Categorical variables were summarized using absolute and relative frequencies, and numerical variables were summarized using means and standard deviations. Cumulative frequencies were used to describe the burden of infections and reinfections throughout the COVID-19 pandemic in Peru.

To estimate the protection conferred by prior infection against reinfection by SARS-CoV-2 variants, we conducted conditional logistic regression analyses for each study period to calculate odds ratios (OR) with 95% confidence intervals (CI) and then converted them into percentage protection using the formula (1-OR) x 100. We additionally made sensitivity analyses adjusting the models by the number of COVID-19 vaccine doses received and assessing protection according to the time since the prior infection (3 to 8 months, 9 to 14 months, ≥15 months). All these analyses were performed using the statistical package Stata version 17 for Windows.

The study was approved by the Research Ethics Committee of the National Institute of Health of Peru. All procedures adhered to ethical standards in accordance with the Declaration of Helsinki.

## RESULTS

During the first study period, 192,843 infections (positive PCR, Antigen, or Serologic test) were recorded, of which 11,583 (4.88%) were possible reinfections (2 positive tests) and 1,223 (0.34%) were probable reinfections (2 positive PCR tests). In the second study period, there were 384,164 infections, with 18,742 (6.01%) possible reinfections and 1,325 (0.63%) probable reinfections. In the third study period, 455,108 infections were registered, with 152,934 (16.0%) possible reinfections and 13,158 (1.19%) probable reinfections. In the fourth study period, 750,514 infections were recorded of which 164,677 (20.4%) were possible reinfections and 15,416 (2.1%) were probable reinfections. For the fifth study period, 1,712,337 infections were recorded, including 274,222 (36.2%) possible reinfections and 20,360 (2.89%) probable reinfections. The detailed dynamics of infection and reinfections during the COVID-19 pandemic in Peru are shown in Figures S1 and S2 of the Supplementary Appendix.

Among the matched case-controls included in the study, the frequency of prior infection was 1,702 (15.0%) in the first study period, 2,003 (16.2%) in the second, 2,071 (20.6%) in the third, 7,298 (30.3%) in the fourth, and 5,201 (40.5%) in the fifth. Demographic characteristics evaluated were well balanced between cases and controls after the matching process, as shown in Tables S3 to S7 (Supplementary Appendix).

The estimated protection conferred by prior infection against SARS-CoV-2 reinfection was 86.3% (95% CI, 81.8 to 89.7) for Lambda, 73.0% (95% CI, 62.9 to 80.3) for Gamma, 85.5% (95% CI, 81.8 to 88.4) for Delta, 34.9% (95% CI, 25.5 to 43.1) for Omicron BA.1, 67.0% (95% CI, 58.7 to 73.6) for Omicron BA.2.12.1, 49.1% (95% CI, 40.5 to 56.5) for Omicron BA.4, 44.8% (95% CI, 39.9 to 49.3) for Omicron BA.5, 29.4% (95% CI, 18.2 to 39.1) for Omicron BQ, and 8.6% (95% CI, -0.5 to 16.9) for Omicron XBB. Protection estimates adjusted by the number of COVID vaccine doses received showed similar results (Table 1).

**Table 1.** Effectiveness of Previous Infection to Prevent SARS-CoV-2 Symptomatic Reinfections by Variants.

Effectiveness to prevent SARS-CoV-2 reinfection	Cases (PCR Positive)		Controls (PCR Negative)		Estimated effectiveness* (95% CI)
	Prior infection	Not prior infection	Prior infection	Not prior infection	
*General analyses*					
SARS-CoV-2 variant					
Lambda	49	1525	1097	5096	86.3 (81.8 to 89.7)
Gamma	44	674	512	2288	73.0 (62.9 to 80.3)
Delta	89	2408	1914	7942	85.5 (81.8 to 88.4)
Omicron BA.1 et al	322	1726	1749	6256	34.9 (25.5 to 43.1)
Omicron BA.2.12.1	87	536	778	1627	67.0 (58.7 to 73.6)
Omicron BA.4 et al	200	751	1177	2455	49.1 (40.5 to 56.5)
Omicron BA.5 et al	778	2660	4278	8733	44.8 (39.9 to 49.3)
Omicron BQ et al	291	462	1227	1493	29.4 (18.2 to 39.1)
Omicron XBB et al	788	1248	2895	4430	8.6 (−0.5 to 16.9)
*Analyses after adjusting by vaccine status*					
SARS-CoV-2 variant					
Lambda	49	1525	1097	5096	85.6 (80.8 to 89.2)
Gamma	44	674	512	2288	71.7 (61.1 to 79.4)
Delta	89	2408	1914	7942	84.9 (81.1 to 87.9)
Omicron BA.1 et al	322	1726	1749	6256	33.9 (24.3 to 42.2)
Omicron BA.2.12.1	87	536	778	1627	67.1 (58.9 to 73.7)
Omicron BA.4 et al	200	751	1177	2455	48.8 (40.1 to 56.2)
Omicron BA.5 et al	778	2660	4278	8733	45.1 (40.2 to 49.5)
Omicron BQ et al	291	462	1227	1493	31.7 (20.8 to 41.2)
Omicron XBB et al	788	1248	2895	4430	10.7 (1.7 to 18.8)

AT: Antigen test, PCR: polymerase chain reaction, NE: not estimable.

* Effectiveness was estimated using a test-negative, nested case-control study design, matching in a 1:4 ratio according to sex, 10-year age group, region of origin, occupation as a health worker, and calendar month of PCR testing. Variant-specific periods were defined as follows: weeks 18 to 34 (2021) for patients infected with the Lambda or Gamma variants; weeks 35 to 51 (2021) for the Delta variant; weeks 52 (2021) to 16 (2022) for Omicron BA.1 et al variants; weeks 17 to 41 (2022) for Omicron BA.2.12.1, BA.4 et al, or BA.5 et al variants; and from weeks 42 (2022) to 6 (2023) for Omicron BQ et al, or XBB et al variants.

**Figure 1.**
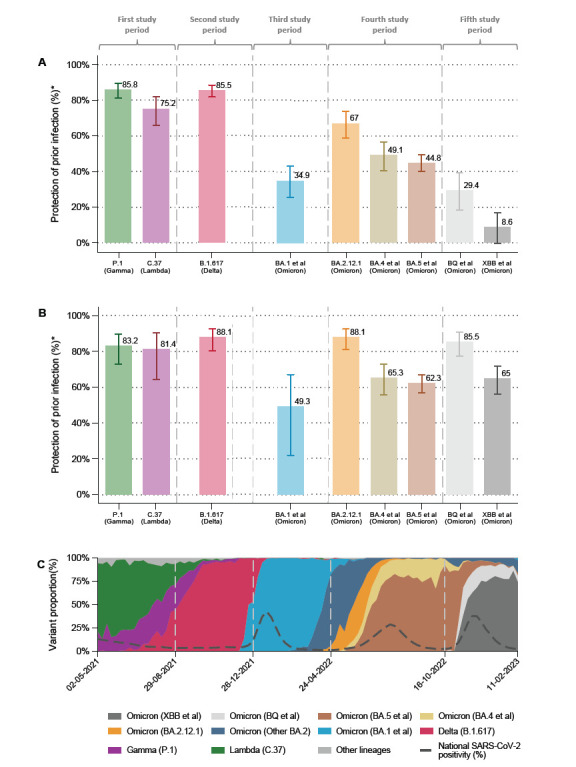
(A) Estimated protection of any time prior infection to prevent reinfections by SARS-CoV-2 variants. (B) Estimated protection of 3 to 8 months prior infection to prevent reinfections by SARS-CoV-2 variants. (C) Proportion of variants during COVID-19 pandemics in Peru.

Regarding the time since prior infection, protection against reinfection after the initial episode was 83.2% (95% CI, 72.8 to 89.6) for Lambda, 81.4% (95% CI, 76.6 to 97.6) for Gamma, 88.1% (95% CI, 80.5 to 92.7) for Delta, 49.3% (95% CI, 22.0 to 67.0) for Omicron BA.1, 88.1% (95% CI, 81.0 to 92.5) for Omicron BA.2.12.1, 65.3% (95% CI, 55.7 to 72.9) for Omicron BA.4, 62.3% (95% CI, 56.9 to 67.0) for Omicron BA.5, 85.5% (95% CI, 77.3 to 90.8) for Omicron BQ, and 65.0% (95% CI, 56.2 to 71.9) for Omicron XBB (Figure 1). Protection estimates for prior infections occurring 9 to 14 months or ≥15 months earlier were similar to those for the 3- to 8-month interval in the case of Lambda, Gamma, Delta, and Omicron BA.1. In contrast, protection was significantly lower for Omicron BA.2.12.1, BA.4, BA.5, BQ, and XBB. Detailed results are presented in Table S8 of the Supplementary Appendix.

*Protection was estimated with a test-negative, nested case–control study design, matching in a 1:4 ratio according to sex, 10-year age group, region of origin, occupation as a health worker, and calendar month of PCR testing. Study periods included week 18 to 34 in 2021 to evaluate infection by Lambda or Gamma variants; weeks 35 to 51 (2021) to evaluate infection by Delta variant; weeks 52 (2021) to 16 (2022) to evaluate infection by Omicron BA.1 et al variant; weeks 17 to 41 (2022) to evaluate infection by Omicron BA.2.12.1, BA.4 et al, or BA.5 et al variants, and weeks 42 (2022) to 6 (2023) to evaluate infection by the Omicron BQ et al, and XBB et al variants.

## DISCUSSION

This study shows that during the COVID-19 pandemic, prior infection provided varying levels of protection against reinfection by SARS-CoV-2. In 2021, prior infection offered high protection (close to 80%) against reinfections caused by Lambda, Gamma, and Delta variants. These findings are consistent with those of Altarawneh et al, who also reported high protection against Delta and other variants that emerged in 2020 and 2021 [6]. This suggests that at that stage of the pandemic, SARS-CoV-2 variants still shared enough similarities to allow memory immune responses (mediated by B and T lymphocytes) and neutralizing antibodies generated by one variant to prevent subsequent infections by others. Furthermore, some studies reported that protection conferred by prior infection during that time was comparable to the protection offered by the primary vaccination scheme based on inactivated SARS-CoV-2 vaccines [12, 13].

On the other hand, between the end of 2021 and start of 2022, we observed a marked decline in protection from prior infection against the Omicron BA.1 sublineage, consistent with the findings of Altarawneh et al in Qatar [6]. This could be attributed to significant mutations in the Spike (S) protein of BA.1 that enable immune evasion from neutralizing antibodies generated by prior infections or vaccination [14]. Supporting this, Carreño et al found reduced neutralization activity against Omicron in serum samples from convalescent (people with prior infection) and vaccinated individuals [15, 16].

At the middle of 2022, prior infection showed a moderate level of protection (50% to 70%) against Omicron BA.2.12.1, BA.4, and BA.5 sublineages. This was lower than observed with earlier variants (Lambda, Gamma, and Delta), despite widespread prior exposure to BA.1 in Peru [17]. Cao et al demonstrated that these Omicron sublineages, while structurally related to BA.1, acquired additional mutations that further enhanced their ability to escape immunity, leading to reinfections even among individuals previously infected with BA.1 [18].

Between late 2022 and early 2023, protection from prior infection dropped to 10% to 30% for Omicron BQ and XBB variants. These findings indicate that these sublineages acquired greater immune escape capabilities than earlier variants, even among individuals previously infected with other Omicron sublineages. Several studies have demonstrated the greater capacity of BQ and XBB to evade the natural immunity produced against BA.4 and BA.5 and bivalent vaccine-induced immunity to these variants [10, 19–21]. However, the specific mutations responsible for this greater immunity evasion are not fully understood, as they share close similarities in the S protein with BA.4 and BA.5 [10].

Our sensitivity analysis focusing on prior infections that occurred 3 to 8 months before testing provides additional insight into the dynamics of cross-variant protection. The findings support the hypothesis that a recent infection is more likely to offer protection against reinfection by a newer variant, especially when both share similar antigenic profiles. In contrast, infections that occurred ≥9 months earlier tend to offer lower protection, possibly due to waning immunity and the accumulation of antigenic changes over time. Notably, for highly divergent Omicron sublineages such as BQ and XBB, protection remained low regardless of whether the prior infection was recent or older, reinforcing their substantial immune escape capacity discussed earlier. This highlights the importance of considering time since prior infection when estimating protection, as failing to do so could lead to misclassification of exposure and underestimate the role of variant similarity in immune-mediated protection.

Our results also help explain the predominance of certain SARS-CoV-2 variants and their relationships with reinfection waves in Peru. For example, the surge in reinfections after the introduction of Omicron BA.1 was substantially greater than in previous waves (See Figure S1, Supplementary Appendix), likely due to the limited protection conferred by prior infection and low vaccination at the time, causing similar patterns to be observed in South Africa [22]. Reinfection waves at the middle of 2022 and late 2022 to early 2023, which correspond to the circulation of BA.4, BA.5, XBB, and BQ, further highlight the role of immune evasion in driving these waves (See Figure S1 and S2, Supplementary Appendix).

Despite these valuable insights, our study has some limitations. First, in Peru, genomic surveillance was applied for people who were evaluated with PCR tests. Thus, our study did not include individuals who were tested only with antigen tests, which, despite being extensively used in Peru to rule out SARS-CoV-2 infections due to their faster turnaround time and acceptable detection performance [23, 24], were not eligible for genomic surveillance. Nevertheless, according to the sanitary regulations provided by the Peruvian MoH, the use of PCR tests was mainly in patients who had reported symptoms within the previous 7 days, reducing the likelihood of obtaining false negative results [25].

Second, the selective inclusion of serologic results, predominantly used in Peru only until the end of 2020, may have introduced classification differences in how “prior infection” was detected, especially compared to subsequent periods in which PCR or antigen tests were the main diagnostic tools. However, since we included mostly symptomatic individuals, the likelihood of missing prior infections during later periods was mitigated; still, we acknowledge the potential for differential bias in early phases. In addition, we used a ≥90-day threshold to define reinfection, in line with standard guidelines. While this approach is widely accepted, it may underestimate rapid reinfections caused by highly transmissible variants like Omicron.

Although we focused on PCR-confirmed cases to define current infection, some variability in test availability and usage, such as antigen tests, may have influenced case detection and sample representativeness across periods. We minimized this by restricting inclusion to PCR-positive results, but residual bias may remain. Similarly, vaccination status was incorporated as the number of doses received, without differentiating vaccine types or defining “full protection.” Given Peru's evolving vaccine strategy, from inactivated vaccines (Sinopharm) to mRNA boosters (Pfizer) and limited bivalent availability, this approach captures general exposure to vaccination but does not account for potential differences in protection by platform.

Differences in non-pharmaceutical interventions could also influence reinfection dynamics. However, the most restrictive measures, such as lockdowns, were implemented in early 2020, outside the study window. Later relaxations, including optional mask use in late 2022, did not coincide with major shifts in reinfection rates (See Figure S2 in Supplementary Appendix), suggesting that immune evasion rather than behavioral changes likely played a more prominent role.

Lastly, the magnitude of effectiveness to prevent reinfections could vary according to the causing variant of previous SARS-CoV-2 infection as mentioned previously in the literature; however, we did not assess these variances by the limitations of the available information. Nevertheless, sensitivity analyses (Table S2, Supplementary Appendix) show the effectiveness to prevent reinfection according to the time from prior infection in delimited periods. This could suggest the potential causing variants of prior infection within these periods considering the 4 to 5 months predominance of the main SARS-CoV-2 variants during the COVID-19 pandemics in Peru.

## CONCLUSIONS

This study confirms that a history of previous infection confers protection against SARS-CoV-2 reinfection [26]. However, this protection varies significantly across variants, with notably lower levels observed against Omicron and its sublineages, regardless of age, sex, and other demographic characteristics. We also observed that protection was higher when the prior infection occurred within the last 3 to 8 months, suggesting that more recent infections retain greater immunologic similarity to newer variants. Infections older than 9 months conferred markedly less protection, especially against highly divergent Omicron sublineages. Future research should evaluate whether prior SARS-CoV-2 infection also reduces the risk of severe outcomes such as hospitalization, ICU admission, and death. In Peru, during the period when Omicron was the predominant variant, active surveillance of infections in unvaccinated individuals was essential, as previous infection alone did not appear to offer sufficient protection against reinfection by newer Omicron sublineages.

## SUPPLEMENTARY DATA

Supplementary materials are available at the *Pathogens and Immunity* website. Supplementary data may be provided by the authors to benefit the reader. Supplementary data are not copyedited and are the sole responsibility of the authors. Questions or comments related to supplementary materials should be addressed to the corresponding author.
