# Supplementary material for: Protection of Prior SARS-CoV-2 Infection Against Different Variants, Including Omicron Descendants, in a Country with High Viral Transmission
Source: Pathog Immun. 2025 May 15;10(2):74–86. doi: 10.20411/pai.v10i2.760 (PMC12087571; doi:10.20411/pai.v10i2.760)
Supplement: Supplementary Tables and Figures [file pai-10-074-s01.pdf]

PATHOGENS AND IMMUNITY
IN ASSOCIATION WITH CASE WESTERN RESERVE UNIVERSITY AND THE RICHARD J. FASENMYER FOUNDATION

Protection of Prior SARS-CoV-2 Infection Against Different Variants, Including Omicron Descendants, in a Country with High Viral Transmission.

Supplementary Appendix

S1. Study Context

S1.1 SARS-CoV-2 reinfections in Peru

In Peru, the COVID-19 pandemics started on March 6, 2020 with the report of the first confirmed SARS-CoV-2 infection (1). Three months later, based on the definition of "probable reinfection" (two positive PCR tests at least 90 days apart), the first cases of SARS-CoV-2 reinfection were reported. Subsequently, in December 2020, more reinfection cases were identified following the incorporation of Antigenic tests (AT) and the adoption of "possible reinfection" definition, which includes two positive results (PCR or AT test), at least 90 days apart (2). As of February 11, 2023, a total of 844,867 cases of possible reinfections had been identified in Peru. Of these, 683,743 (80.9%) has one reinfection episode, 157,189 (18.6%) involved two episodes, 3,760 (0.45%) involved three, 170 (0.02%) involved four, and 5 cases (<0.01%) involved five reinfection episodes. Figure S1 and S2 showed the daily and cumulative frequencies of infections and reinfections since the start of random genomic surveillance in Peru. A higher increase in reinfections was observed during the third wave (predominantly Omicron BA.1) variant, fourth wave (Omicron BA.4 and BA.5), and the fifth wave (Omicron BQ and XBB variants) (see S1.2 SARS-CoV-2 variants in Peru).

Figure S1. Daily cases and reinfections cases of SARS-CoV-2 in Peru, 2021-2023.

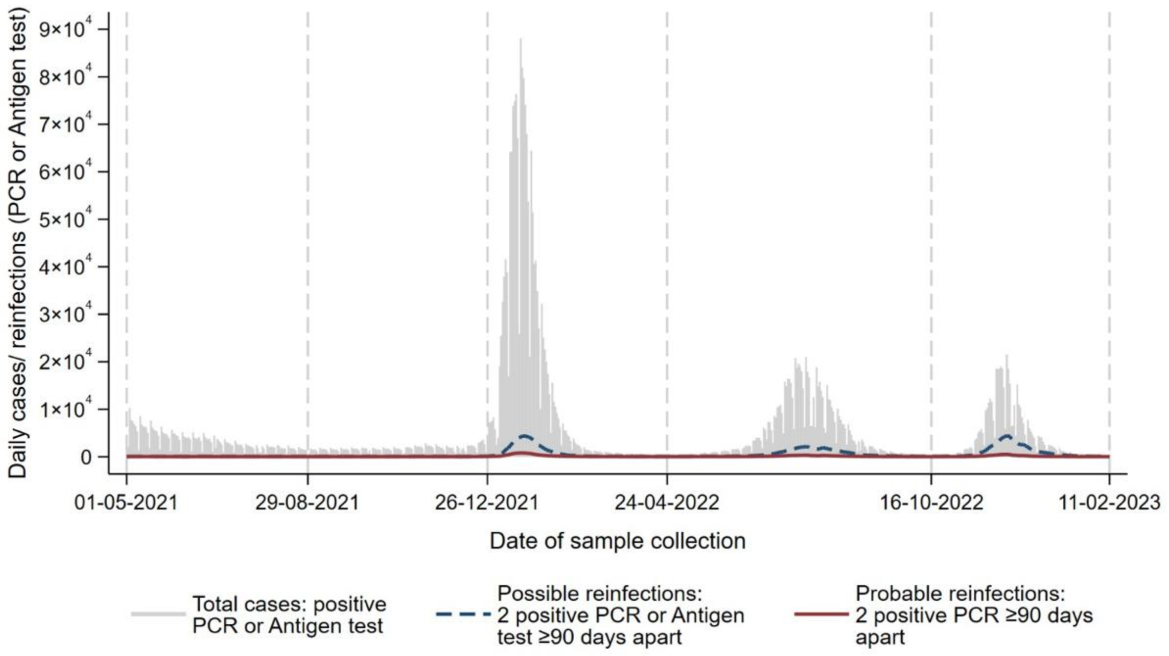

(---) Gray dashed lines indicates the limits of the four periods of study

Escobar-Agreda S, Araujo-Castillo RV, Pampa-Espinoza L, Silva-Valencia J, Solari L. Protection of Prior SARS-CoV-2 Infection Against Different Variants, Including Omicron Descendants, in a Country with High Viral Transmission. *Pathogens and Immunity*. 2025;10(2):74–86. doi: 10.20411/pai.v10i2.760

Figure S2. Cumulative cases and reinfections cases of SARS-CoV-2 in Peru, 2021-2023.

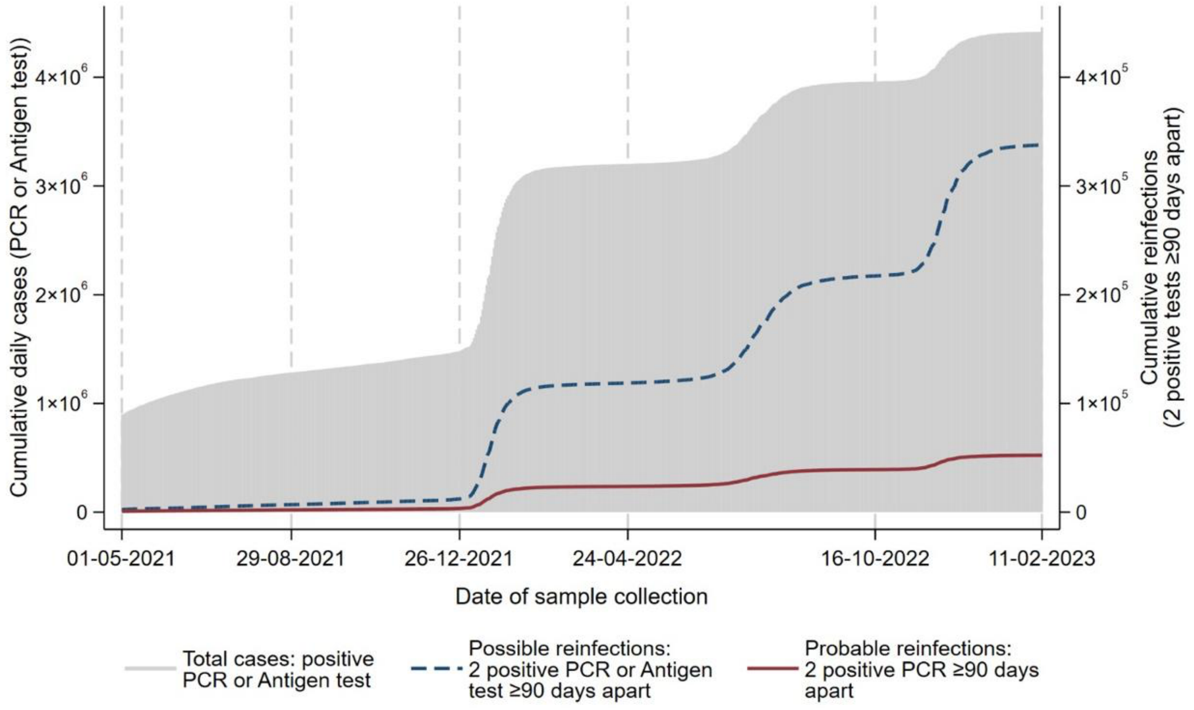

(---) Gray dashed lines indicates the limits of the four study periods.

S1.2. SARS-CoV-2 variants in Peru

Giving the emergence of different SARS-CoV-2 lineages and sub-linages during the COVID-19 pandemic, the Peruvian Ministry of Health, through de Peruvian National Institute of Health (INS in Spanish) deployed a Genomic SARS-CoV-2 surveillance system to identify the variants of concern (VOC) and variants of interest (VOI), due to their public health relevance (3). Sequencing SARS-CoV-2 samples in Peru began in December 2020. However, starting on April 25, 2021, a random selection strategy was adopted to improve representativeness. A total of 4,477 samples were sequenced between that date and September 10, 2022. According to Figure 1 the predominant variants found were C.37 (Lambda) and P.1 (Gamma) in the second third of 2021, B.1.617.2 (Delta) on the last third of 2021, Omicron BA.1 et al in the first third of 2022, and Omicron BA.2.12.1, BA.4 and BA.5 et al in the second third of 2022. Other identified variants included some VOCs such as B.1.1.7 (Alpha), and VOIs such as B.1.525 (Eta), B.1.526 (Iota), as well as recombinant lineages (see Table S1). Figure S3 shows the distribution of predominant variants over the Peruvian macro-regions. One notable difference is the predominance of P.1 (Gamma) over C.37 (Lambda) during the second third of 2021 in the Amazon rainforest macro-region, a phenomenon previously reported in the literature (4).

Escobar-Agreda S, Araujo-Castillo RV, Pampa-Espinoza L, Silva-Valencia J, Solari L. Protection of Prior SARS-CoV-2 Infection Against Different Variants, Including Omicron Descendants, in a Country with High Viral Transmission. *Pathogens and Immunity*. 2025;10(2):74–86. doi: 10.20411/pai.v10i2.760

Table S1. SARS-CoV-2 variants during COVID-19 pandemic in Peru, 2021-2023.

Variant	n	%
Alpha	16	0.04
Delta	5,967	14.05
European B.1	324	0.76
Gamma	1,913	4.5
Lambda	3,888	9.15
Mu	257	0.61
Omicron BA.1 et al	4,783	11.26
Omicron BA.2.12.1 et al	1,963	4.62
Omicron BA.4 et al	2,695	6.35
Omicron BA.5 et al	10,619	25
Omicron BQ et al	1,716	4.04
Omicron XBB et al	5,906	13.91
Other Omicron BA.2	2,392	5.63
Recombinant	29	0.07
Zeta	1	0

*Includes Eta, Iota, and other derivatives sub-lineages from B.1

Figure S3. Proportion of SARS-CoV-2 variants over macro regions. Peru, 2021-2023.

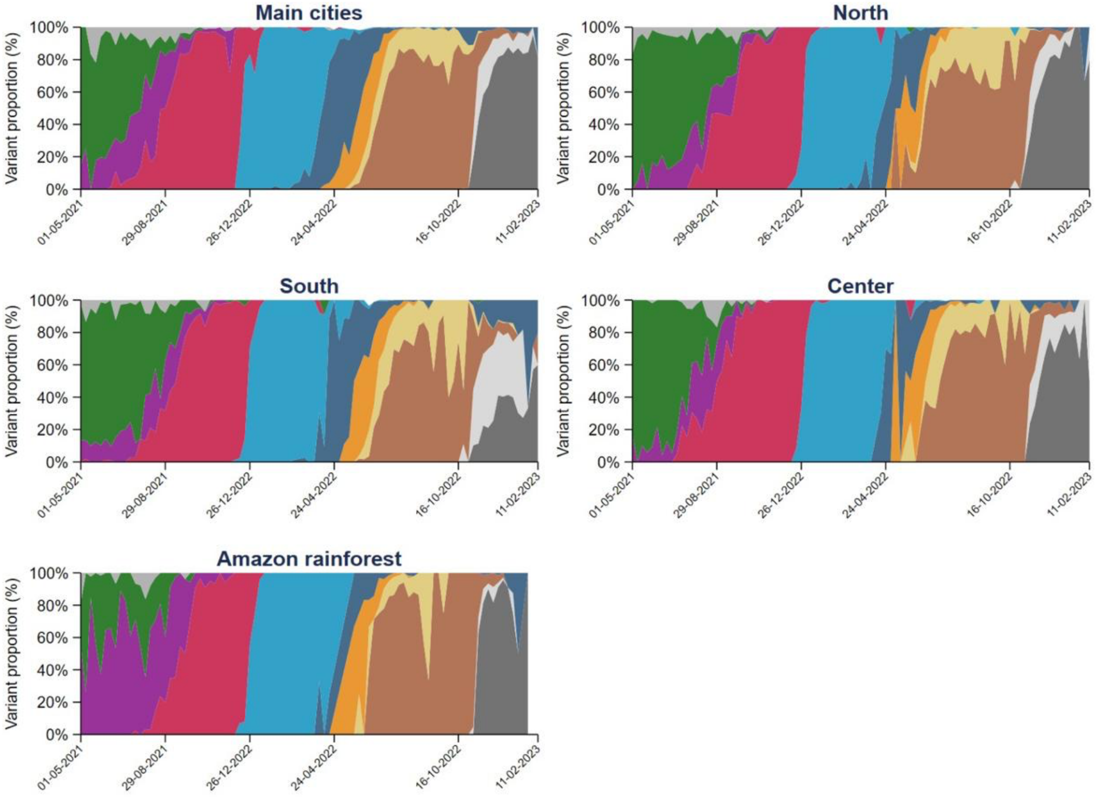

*Main cities included Lima and Callao.

Escobar-Agreda S, Araujo-Castillo RV, Pampa-Espinoza L, Silva-Valencia J, Solari L. Protection of Prior SARS-CoV-2 Infection Against Different Variants, Including Omicron Descendants, in a Country with High Viral Transmission. *Pathogens and Immunity*. 2025;10(2):74–86. doi: 10.20411/pai.v10i2.760

Table S2. Variables included in the study.

Variable	Values	Sources
Time to infection / censorship	[0-126] days	Information System of the National Network of Public Health Laboratories in Perú (NETLAB)
Indicator of laboratory-confirmed SARS-CoV-2 infection (‡)	0 = Non infected<br>1 = Infected	Information System of the National Network of Public Health Laboratories in Perú (NETLAB) and Integrated System COVID-19 (SICOVID).
Indicator of laboratory-confirmed<br>SARS-CoV-2 identified variant	First study period:<br>0 = Non infected<br>1 = Lambda<br>2 = Gamma<br><br>Second study period:<br>0 = Non infected<br>1 = Delta<br><br>Third study period:<br>0 = Non infected<br>1 = Omicron BA.1 et al<br><br>Fourth study period:<br>0 = Non infected<br>1 = Omicron BA.2.12.1<br>2 = Omicron BA.4 et al<br>3 = Omicron BA.5 et al	Information System of the National Network of Public Health Laboratories in Perú (NETLAB).
Previous SARS-CoV-2 infection	0 = No<br>1 = Yes	Information System of the National Network of Public Health Laboratories in Perú (NETLAB) and Integrated System COVID-19 (SICOVID).
Vaccination status	0 = Zero doses<br>1 = One dose<br>2 = Two doses<br>3 = Threes doses<br>4 = Four doses<br>5 = Five doses	National vaccination registry of the Peruvian Ministry of Health.
Sex	0 = Female<br>1 = Male	Information System of the National Network of Public Health Laboratories in Perú (NETLAB).
Age group	1 = 0-9 years<br>2 = 10-19 years<br>3 = 20-29 years<br>4 = 30-39 years<br>5 = 40-49 years<br>6 = 50-59 years<br>7 = 60-69 years<br>8 = 70-79 years<br>9 = 80-89 years<br>10 = 90-100 years	Information System of the National Network of Public Health Laboratories in Perú (NETLAB).

Escobar-Agreda S, Araujo-Castillo RV, Pampa-Espinoza L, Silva-Valencia J, Solari L. Protection of Prior SARS-CoV-2 Infection Against Different Variants, Including Omicron Descendants, in a Country with High Viral Transmission. *Pathogens and Immunity*. 2025;10(2):74–86. doi: 10.20411/pai.v10i2.760

Region of origin	1 = Center<br>2 = Lima-Callao<br>3 = North<br>4 = East<br>5 = South	Information System of the National Network of Public Health Laboratories in Perú (NETLAB).
Health worker status	0 = non-Health Worker<br>1 = Health Worker	Ministry of Health vaccination registry of health care workers

Figure S4. Information source matching flow diagram to obtain the final database

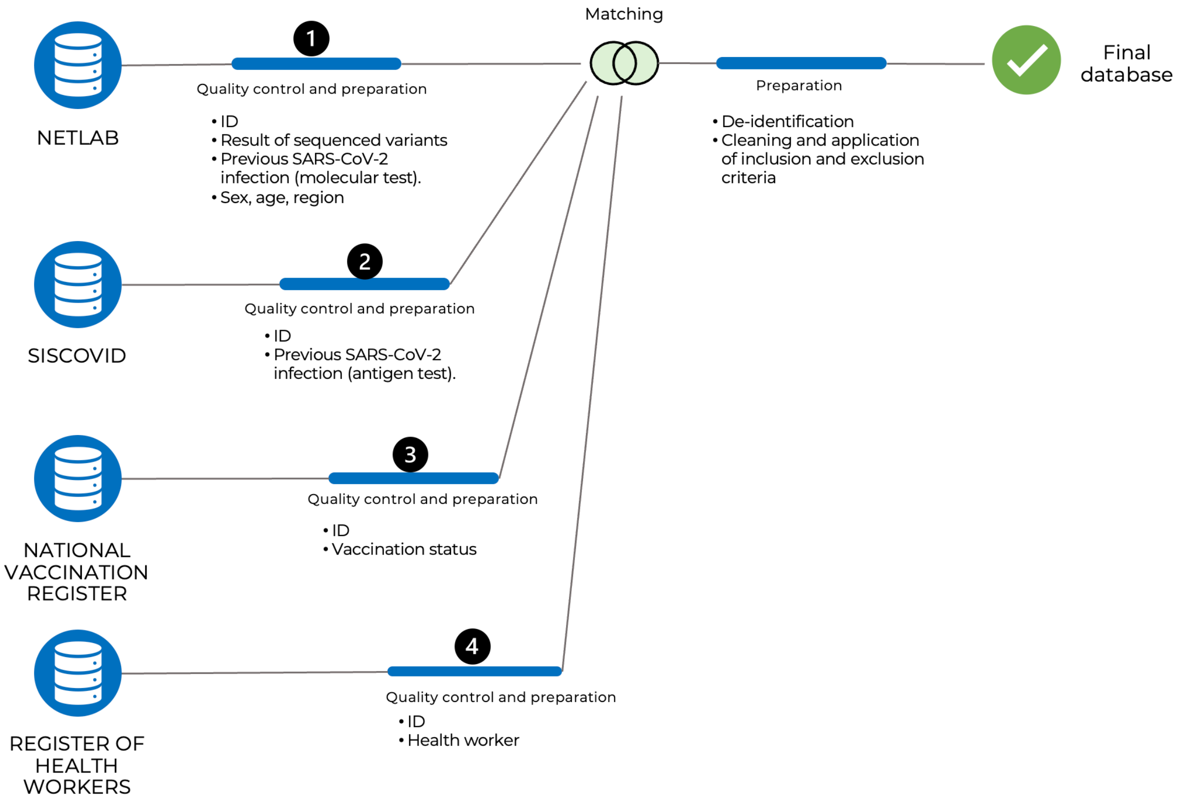

S3. Laboratory methods

Nasopharyngeal swabs were transported in viral transport media and homogenized by vortexing prior ARN extraction. Genetic material was automatically extracted with Auto-Pure 96 Allsheng equipment to ensure ARN quality, concentration, and integrity. The presence of SARS-CoV-2 was tested by real-time reverse-transcription PCR (RT-qPCR) using SARS-CoV-2 Nucleic Acid Detection Kit (Lifotronic), which targets the ORF1ab and N viral gene regions, as well as a fragment of human RNase P gene as internal control. Reactions were performed using Molarray MA6000 thermal cyclers. Samples with a cycle treshold (Ct) value < 30 were selected for genomic sequencing.

Positive samples were randomly selected by epidemiological week from all Peruvian Regions, processed by Illumina COVISEQ kit, and sequenced in NextSeq 550 platform. Genome assembly was performed by mapping with reference Wuhan-Hu-1 (GenBank accession NC_045512.2) using Samtools 1.9 and iVar 1.3.1. Lineage identification was conducted using PANGOLIN algorithm, followed by phylogenetic reconstruction with RAxML under the GTR+I+G substitution model, using 20 independent parsimony starting trees. Aditionally, SARS-CoV-2 variant-diagnostic-mutations were identified using Nextclade (https://clades.nextstrain.org).

Figure S5. Flowchart of the selection process for the first study period to assess the effectiveness of prior SARS-CoV-2 infection in preventing reinfection with the Lambda or Gamma variants.

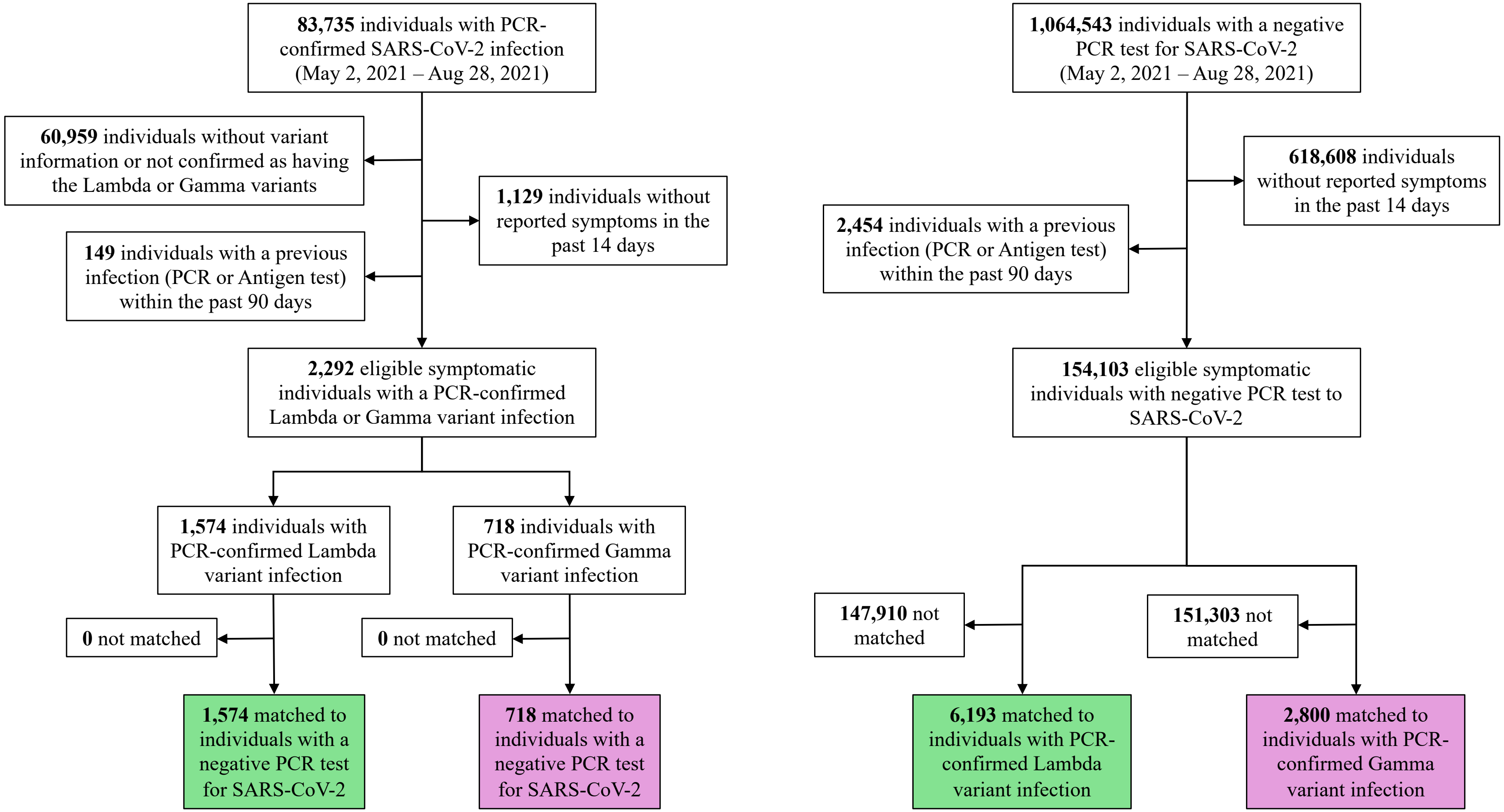

PCR: Polymerase chain reaction test (molecular test)
Cases were matched with controls in a 4 to 1 ratio by sex, 10-year age group, macro-region of origin, being a or not a health worker and the PCR test week for the first eligible person with a negative PCR test to SARS-CoV-2.

Escobar-Agreda S, Araujo-Castillo RV, Pampa-Espinoza L, Silva-Valencia J, Solari L. Protection of Prior SARS-CoV-2 Infection Against Different Variants, Including Omicron Descendants, in a Country with High Viral Transmission. *Pathogens and Immunity*. 2025;10(2):74–86. doi: 10.20411/pai.v10i2.760

Figure S6. Flowchart of the selection process for the second study period to assess the effectiveness of prior SARS-CoV-2 infection in preventing reinfection with the Delta variant.

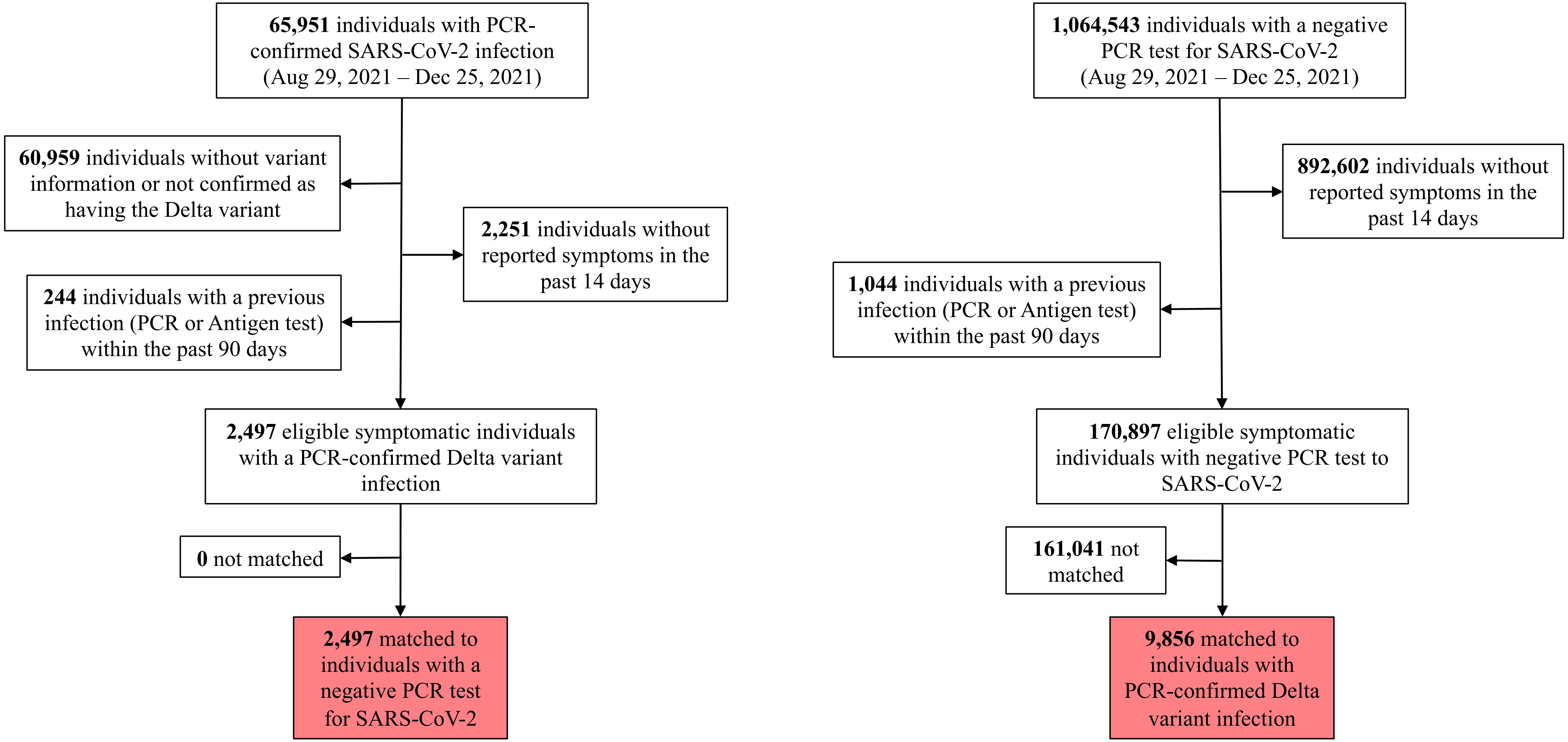

PCR: Polymerase chain reaction test (molecular test)
Cases were matched with controls in a 4 to 1 ratio by sex, 10-year age group, macro-region of origin, being a or not a health worker and the PCR test week for the first eligible person with a negative PCR test to SARS-CoV-2.

Escobar-Agreda S, Araujo-Castillo RV, Pampa-Espinoza L, Silva-Valencia J, Solari L. Protection of Prior SARS-CoV-2 Infection Against Different Variants, Including Omicron Descendants, in a Country with High Viral Transmission. *Pathogens and Immunity*. 2025;10(2):74–86. doi: 10.20411/pai.v10i2.760

Figure S7. Flowchart of the selection process for the third study period to assess the effectiveness of prior SARS-CoV-2 infection in preventing reinfection with the Omicron BA.1 variant.

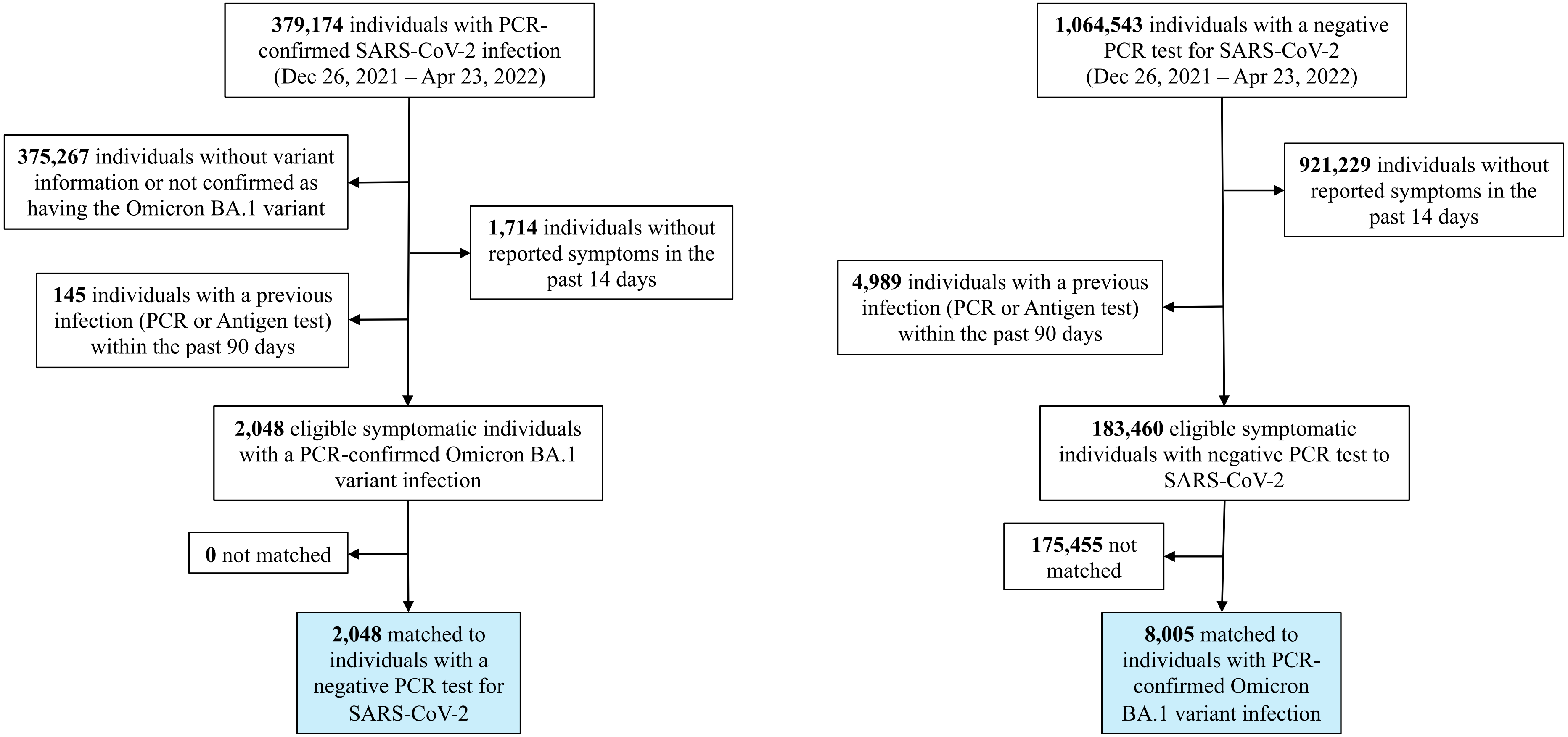

PCR: Polymerase chain reaction test (molecular test)
Cases were matched with controls in a 4 to 1 ratio by sex, 10-year age group, macro-region of origin, being a or not a health worker and the PCR test week for the first eligible person with a negative PCR test to SARS-CoV-2.

Escobar-Agreda S, Araujo-Castillo RV, Pampa-Espinoza L, Silva-Valencia J, Solari L. Protection of Prior SARS-CoV-2 Infection Against Different Variants, Including Omicron Descendants, in a Country with High Viral Transmission. *Pathogens and Immunity*. 2025;10(2):74–86. doi: 10.20411/pai.v10i2.760

PATHOGENS AND IMMUNITY
IN ASSOCIATION WITH CASE WESTERN RESERVE UNIVERSITY AND THE RICHARD J. FASENMYER FOUNDATION

Figure S8. Flowchart of the selection process for the fourth study period to assess the effectiveness of prior SARS-CoV-2 infection in preventing reinfection with the Omicron BA.2.12.1, BA.4 or BA.5 et al variants.

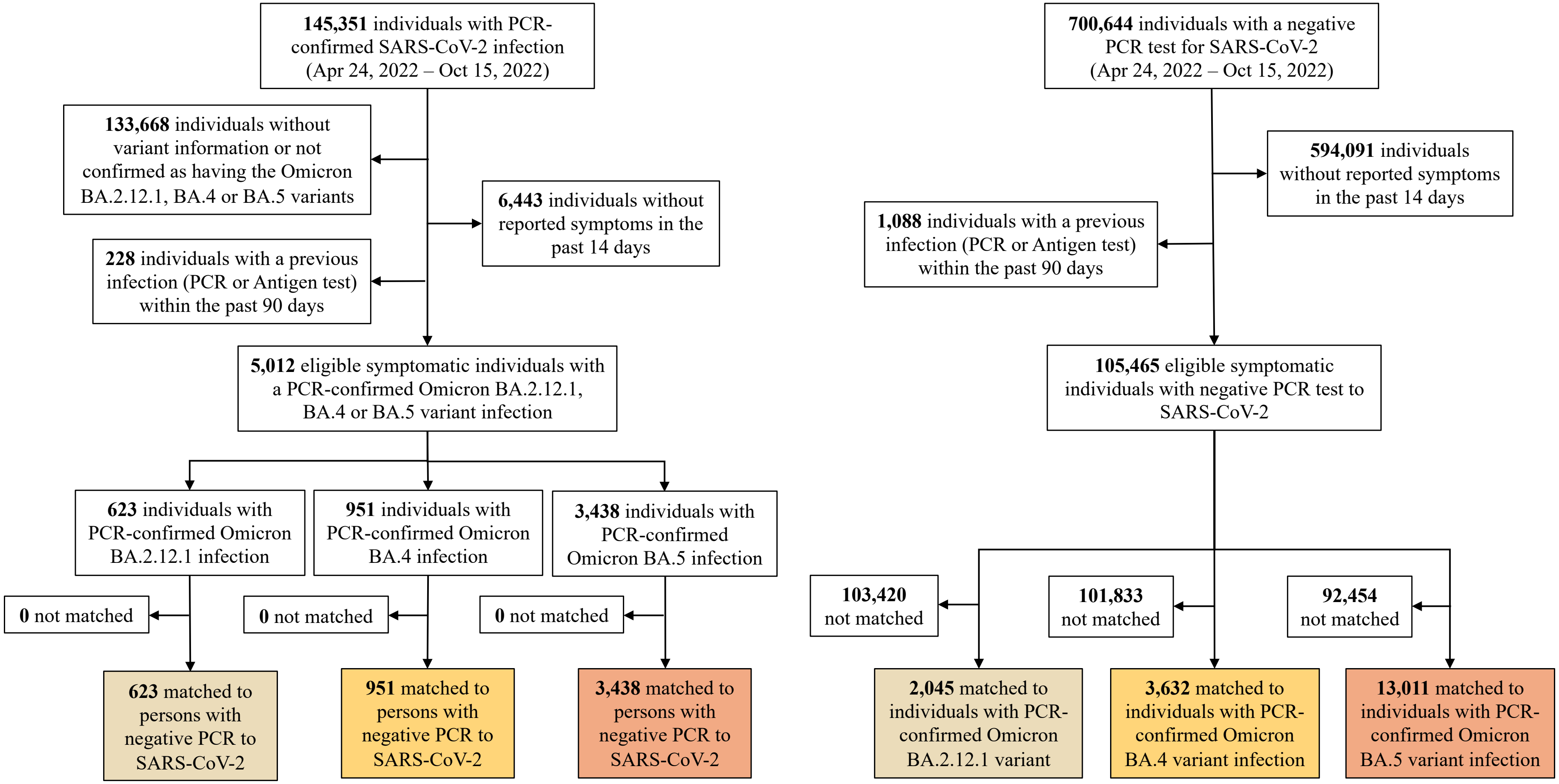

PCR: Polymerase chain reaction test (molecular test)
Cases were matched with controls in a 4 to 1 ratio by sex, 10-year age group, macro-region of origin, being a or not a health worker and the PCR test week for the first eligible person with a negative PCR test to SARS-CoV-2.

Escobar-Agreda S, Araujo-Castillo RV, Pampa-Espinoza L, Silva-Valencia J, Solari L. Protection of Prior SARS-CoV-2 Infection Against Different Variants, Including Omicron Descendants, in a Country with High Viral Transmission. *Pathogens and Immunity*. 2025;10(2):74–86. doi: 10.20411/pai.v10i2.760

Figure S9. Flowchart of the selection process for the fifth study period to assess the effectiveness of prior SARS-CoV-2 infection in preventing reinfection with the Omicron BQ or XBB variants.

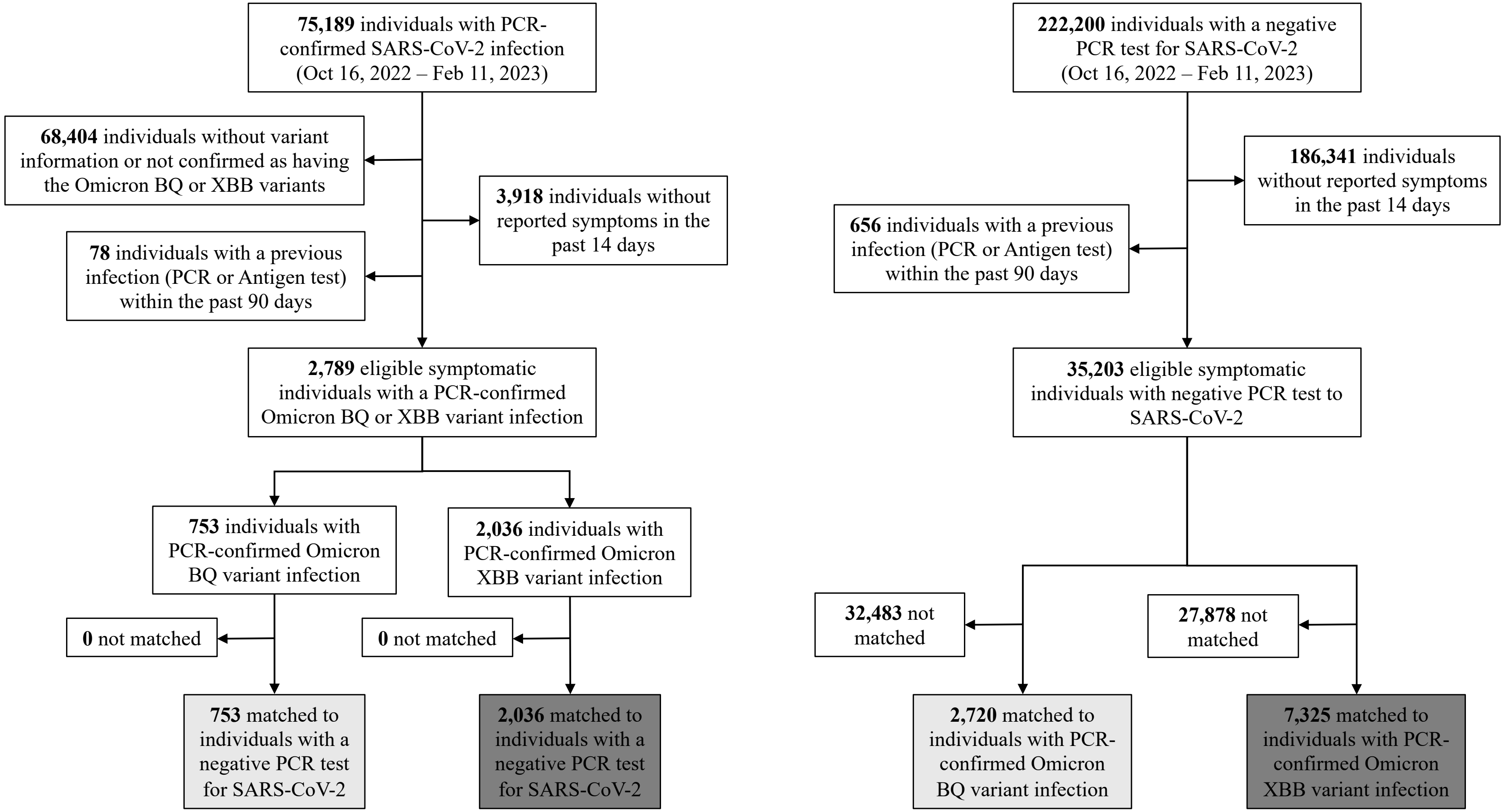

PCR: Polymerase chain reaction test (molecular test)
Cases were matched with controls in a 4 to 1 ratio by sex, 10-year age group, macro-region of origin, being a or not a health worker and the PCR test week for the first eligible person with a negative PCR test to SARS-CoV-2.

Escobar-Agreda S, Araujo-Castillo RV, Pampa-Espinoza L, Silva-Valencia J, Solari L. Protection of Prior SARS-CoV-2 Infection Against Different Variants, Including Omicron Descendants, in a Country with High Viral Transmission. *Pathogens and Immunity*. 2025;10(2):74–86. doi: 10.20411/pai.v10i2.760

Table S3. Characteristics of matched cases (PCR-positive persons to Lambda or Gamma variants) and controls (PCR-negative persons) from the first study period.

	Lambda Cases (PCR-Positive)		Lambda Controls (PCR-Negative)		SMD†	Gamma Cases (PCR-Positive)		Gamma Controls (PCR-Negative)		SMD†
	Nro.	(%)	Nro.	(%)		Nro.	(%)	Nro.	(%)	
**Total**	1574	(100)	6193	(100)		718	(100)	2800	(100)	
**Sex**										
Female	836	(53.1)	3302	(53.3)	<0.01	371	(51.7)	1461	(52.2)	0.01
Male	738	(46.9)	2891	(46.7)		347	(48.3)	1339	(47.8)	
**Categorized age (10 years)**										
0-9 years	19	(1.2)	64	(1.0)	0.04	8	(1.1)	31	(1.1)	0.07
10-19 years	151	(9.6)	597	(9.6)		53	(7.4)	211	(7.5)	
20-29 years	303	(19.3)	1206	(19.5)		148	(20.6)	582	(20.8)	
30-39 years	362	(23.0)	1441	(23.3)		167	(23.3)	667	(23.8)	
40-49 years	309	(19.6)	1221	(19.7)		142	(19.8)	554	(19.8)	
50-59 years	251	(15.9)	999	(16.1)		118	(16.4)	465	(16.6)	
60-69 years	104	(6.6)	402	(6.5)		55	(7.7)	215	(7.7)	
70-79 years	48	(3.0)	179	(2.9)		21	(2.9)	63	(2.3)	
80-89 years	21	(1.3)	72	(1.2)		5	(0.7)	11	(0.4)	
90-100 years	6	(0.4)	12	(0.2)		1	(0.1)	1	(0.0)	
**Macro-region**										
Lima-Callao	365	(23.2)	1450	(23.4)	0.01	226	(31.5)	893	(31.9)	0.01
North	212	(13.5)	824	(13.3)		55	(7.7)	217	(7.8)	
South	628	(39.9)	2497	(40.3)		110	(15.3)	428	(15.3)	
Center	227	(14.4)	868	(14.0)		71	(9.9)	268	(9.6)	
West	142	(9.0)	554	(8.9)		256	(35.7)	994	(35.5)	
**Health worker**										

Escobar-Agreda S, Araujo-Castillo RV, Pampa-Espinoza L, Silva-Valencia J, Solari L. Protection of Prior SARS-CoV-2 Infection Against Different Variants, Including Omicron Descendants, in a Country with High Viral Transmission. *Pathogens and Immunity*. 2025;10(2):74–86. doi: 10.20411/pai.v10i2.760

Not health worker	1449	(92.1)	5732	(92.6)	0.02	644	(89.7)	2543	(90.8)	0.04
Health worker	125	(7.9)	461	(7.4)		74	(10.3)	257	(9.2)	
**Epidemiological week (2021)**										
18th week	73	(4.6)	289	(4.7)		23	(3.2)	88	(3.1)	
19th week	69	(4.4)	276	(4.5)		15	(2.1)	60	(2.1)	
20th week	66	(4.2)	264	(4.3)		45	(6.3)	173	(6.2)	
21st week	45	(2.9)	176	(2.8)		9	(1.3)	36	(1.3)	
22rd week	225	(14.3)	896	(14.5)		48	(6.7)	191	(6.8)	
23th week	199	(12.6)	792	(12.8)		67	(9.3)	258	(9.2)	
24th week	149	(9.5)	582	(9.4)		43	(6.0)	169	(6.0)	
25th week	85	(5.4)	326	(5.3)		25	(3.5)	96	(3.4)	
26th week	68	(4.3)	260	(4.2)	0.01	21	(2.9)	84	(3.0)	0.02
27th week	129	(8.2)	504	(8.1)		73	(10.2)	280	(10.0)	
28th week	141	(9.0)	550	(8.9)		63	(8.8)	249	(8.9)	
29th week	96	(6.1)	383	(6.2)		55	(7.7)	216	(7.7)	
30th week	38	(2.4)	147	(2.4)		27	(3.8)	102	(3.6)	
31st week	58	(3.7)	227	(3.7)		51	(7.1)	199	(7.1)	
32nd week	73	(4.6)	286	(4.6)		55	(7.7)	214	(7.6)	
33rd week	37	(2.4)	146	(2.4)		59	(8.2)	236	(8.4)	
34th week	23	(1.5)	89	(1.4)		39	(5.4)	149	(5.3)	
34th week	23	(1.5)	89	(1.4)		39	(5.4)	149	(5.3)	

PCR, polymerase chain reaction; SMD, standardized mean difference.
*Cases and controls were exact matched one-to-four by sex, 10-year age group, macro-region, health worker status and epidemiological week of PCR test.
†SMD is the difference in the mean of a covariate between groups divided by the pooled standard deviation.

Escobar-Agreda S, Araujo-Castillo RV, Pampa-Espinoza L, Silva-Valencia J, Solari L. Protection of Prior SARS-CoV-2 Infection Against Different Variants, Including Omicron Descendants, in a Country with High Viral Transmission. *Pathogens and Immunity*. 2025;10(2):74–86. doi: 10.20411/pai.v10i2.760

PATHOGENS AND IMMUNITY
IN ASSOCIATION WITH CASE WESTERN RESERVE UNIVERSITY AND THE RICHARD J. FASENMYER FOUNDATION

Table S4. Characteristics of matched cases (PCR-positive persons to Delta variant) and controls (PCR-negative persons) from the second study period.

	Delta				
	Cases (PCR-Positive)		Controls (PCR-Negative)		SMD†
	Nro.	(%)	Nro.	(%)	
**Total**	2497	(100)	9856	(100)	
**Sex**					
Female	1287	(51.5)	5109	(51.8)	<0.01
Male	1210	(48.5)	4747	(48.2)	
**Categorized age (10 years)**					
0-9 years	44	(1.8)	169	(1.7)	0.04
10-19 years	248	(9.9)	980	(9.9)	
20-29 years	584	(23.4)	2332	(23.7)	
30-39 years	570	(22.8)	2272	(23.1)	
40-49 years	388	(15.5)	1545	(15.7)	
50-59 years	306	(12.3)	1207	(12.2)	
60-69 years	187	(7.5)	730	(7.4)	
70-79 years	111	(4.4)	422	(4.3)	
80-89 years	50	(2.0)	181	(1.8)	
90-100 years	9	(0.4)	18	(0.2)	
**Macro-region**					
Lima-Callao	673	(27.0)	2685	(27.2)	<0.01
North	671	(26.9)	2654	(26.9)	
South	513	(20.5)	2025	(20.5)	
Center	459	(18.4)	1794	(18.2)	
West	181	(7.2)	698	(7.1)	
**Health worker**					
Not health worker	2328	(93.2)	9227	(93.6)	0.01
Health worker	169	(6.8)	629	(6.4)	
**Epidemiological week (2021)**					
35th week	55	(2.2)	213	(2.2)	<0.01
36th week	90	(3.6)	355	(3.6)	
37th week	92	(3.7)	359	(3.6)	
38th week	130	(5.2)	514	(5.2)	
39th week	121	(4.8)	473	(4.8)	
40th week	121	(4.8)	481	(4.9)	
41st week	189	(7.6)	739	(7.5)	
42nd week	190	(7.6)	751	(7.6)	
43rd week	158	(6.3)	624	(6.3)	
44th week	153	(6.1)	605	(6.1)	
45th week	194	(7.8)	769	(7.8)	
46th week	182	(7.3)	722	(7.3)	
47th week	172	(6.9)	686	(7.0)	

Escobar-Agreda S, Araujo-Castillo RV, Pampa-Espinoza L, Silva-Valencia J, Solari L. Protection of Prior SARS-CoV-2 Infection Against Different Variants, Including Omicron Descendants, in a Country with High Viral Transmission. *Pathogens and Immunity*. 2025;10(2):74–86. doi: 10.20411/pai.v10i2.760

48th week	145	(5.8)	567	(5.8)	
49th week	219	(8.8)	863	(8.8)	
50th week	178	(7.1)	708	(7.2)	
51st week	108	(4.3)	427	(4.3)	

PCR, polymerase chain reaction; SMD, standardized mean difference.
*Cases and controls were exact matched one-to-four by sex, 10-year age group, macro-region, health worker status and epidemiological week of PCR test.
†SMD is the difference in the mean of a covariate between groups divided by the pooled standard deviation.

Table S5. Characteristics of matched cases (PCR-positive persons to Omicron BA.1 et al variant) and controls (PCR-negative persons) from the third study period.

	Omicron BA.1 et al				
	Cases (PCR-Positive)		Controls (PCR-Negative)		SMD†
	Nro.	(%)	Nro.	(%)	
**Total**	2048	(100)	8005	(100)	
**Sex**					
Female	1191	(58.2)	4676	(58.4)	<0.01
Male	857	(41.8)	3329	(41.6)	
**Categorized age (10 years)**					
0-9 years	36	(1.8)	141	(1.8)	
10-19 years	189	(9.2)	753	(9.4)	
20-29 years	383	(18.7)	1510	(18.9)	
30-39 years	430	(21.0)	1710	(21.4)	
40-49 years	344	(16.8)	1366	(17.1)	0.04
50-59 years	276	(13.5)	1082	(13.5)	
60-69 years	201	(9.8)	781	(9.8)	
70-79 years	100	(4.9)	374	(4.7)	
80-89 years	69	(3.4)	256	(3.2)	
90-100 years	20	(1.0)	32	(0.4)	
**Macro-region**					
Lima-Callao	191	(9.3)	735	(9.2)	
North	486	(23.7)	1904	(23.8)	
South	646	(31.5)	2542	(31.8)	<0.01
Center	391	(19.1)	1539	(19.2)	
West	334	(16.3)	1285	(16.1)	
**Health worker**					
Not health worker	1782	(87.0)	7041	(88.0)	0.02
Health worker	266	(13.0)	964	(12.0)	
**Epidemiological week (2021-2022)**					
52nd week	54	(2.6)	211	(2.6)	
1st week	231	(11.3)	920	(11.5)	0.02
2nd week	170	(8.3)	676	(8.4)	
3rd week	185	(9.0)	737	(9.2)	

Escobar-Agreda S, Araujo-Castillo RV, Pampa-Espinoza L, Silva-Valencia J, Solari L. Protection of Prior SARS-CoV-2 Infection Against Different Variants, Including Omicron Descendants, in a Country with High Viral Transmission. *Pathogens and Immunity*. 2025;10(2):74–86. doi: 10.20411/pai.v10i2.760

4th week	206	(10.1)	812	(10.1)
5th week	283	(13.8)	1101	(13.8)
6th week	214	(10.4)	829	(10.4)
7th week	185	(9.0)	722	(9.0)
8th week	163	(8.0)	627	(7.8)
9th week	112	(5.5)	440	(5.5)
10th week	93	(4.5)	355	(4.4)
11th week	75	(3.7)	285	(3.6)
12th week	37	(1.8)	146	(1.8)
13th week	17	(0.8)	60	(0.7)
14th week	12	(0.6)	44	(0.5)
15th week	5	(0.2)	16	(0.2)
16th week	6	(0.3)	24	(0.3)

PCR, polymerase chain reaction; SMD, standardized mean difference.
*Cases and controls were exact matched one-to-four by sex, 10-year age group, macro-region, health worker status and epidemiological week of PCR test.
†SMD is the difference in the mean of a covariate between groups divided by the pooled standard deviation.

Escobar-Agreda S, Araujo-Castillo RV, Pampa-Espinoza L, Silva-Valencia J, Solari L. Protection of Prior SARS-CoV-2 Infection Against Different Variants, Including Omicron Descendants, in a Country with High Viral Transmission. *Pathogens and Immunity*. 2025;10(2):74–86. doi: 10.20411/pai.v10i2.760

Table S6. Characteristics of matched cases (PCR-positive persons to Omicron BA.2.12.1, Omicron BA.4 et al or Omicron BA.5 et al variants) and controls (PCR-negative persons) from the fourth study period.

	Omicron BA.2.12.1					Omicron BA.4 et al					Omicron BA.5 et al				
	Cases (PCR-Positive)		Controls (PCR-Negative)		SMD†	Cases (PCR-Positive)		Controls (PCR-Negative)		SMD†	Cases (PCR-Positive)		Controls (PCR-Negative)		SMD†
	Nro.	(%)	Nro.	(%)		Nro.	(%)	Nro.	(%)		Nro.	(%)	Nro.	(%)	
**Total**	623	(100)	2405	(100)		951	(100)	3632	(100)		3438	(100)	13011	(100)	
**Sex**															
Female	373	(59.9)	1459	(60.7)	0.02	565	(59.4)	2172	(59.8)	<0.01	2012	(58.5)	7668	(58.9)	<0.01
Male	250	(40.1)	946	(39.3)		386	(40.6)	1460	(40.2)		1426	(41.5)	5343	(41.1)	
**Categorized age (10 years)**															
0-9 years	8	(1.3)	29	(1.2)	0.07	27	(2.8)	103	(2.8)	0.04	79	(2.3)	283	(2.2)	0.08
10-19 years	58	(9.3)	231	(9.6)		111	(11.7)	439	(12.1)		397	(11.6)	1565	(12.0)	
20-29 years	96	(15.4)	374	(15.6)		158	(16.6)	620	(17.1)		617	(18.0)	2382	(18.3)	
30-39 years	121	(19.4)	479	(19.9)		197	(20.7)	775	(21.3)		658	(19.1)	2568	(19.7)	
40-49 years	119	(19.1)	464	(19.3)		175	(18.4)	666	(18.3)		596	(17.3)	2302	(17.7)	
50-59 years	96	(15.4)	369	(15.3)		139	(14.6)	525	(14.5)		504	(14.7)	1914	(14.7)	
60-69 years	76	(12.2)	283	(11.8)		93	(9.8)	326	(9.0)		350	(10.2)	1254	(9.6)	
70-79 years	37	(5.9)	140	(5.8)		33	(3.5)	120	(3.3)		145	(4.2)	488	(3.8)	
80-89 years	9	(1.4)	33	(1.4)		14	(1.5)	47	(1.3)		81	(2.4)	249	(1.9)	
90-100 years	3	(0.5)	3	(0.1)		4	(0.4)	11	(0.3)		10	(0.3)	6	(0.0)	
**Macro-region**															
Lima-Callao	194	(31.1)	760	(31.6)	0.03	100	(10.5)	391	(10.8)	0.02	349	(10.2)	1343	(10.3)	0.02
North	81	(13.0)	308	(12.8)		164	(17.2)	630	(17.3)		723	(21.0)	2738	(21.0)	
South	217	(34.8)	849	(35.3)		340	(35.8)	1324	(36.5)		830	(24.1)	3192	(24.5)	
Center	81	(13.0)	292	(12.1)		239	(25.1)	886	(24.4)		590	(17.2)	2157	(16.6)	
West	50	(8.0)	196	(8.1)		108	(11.4)	401	(11.0)		946	(27.5)	3581	(27.5)	
**Health worker**															

Escobar-Agreda S, Araujo-Castillo RV, Pampa-Espinoza L, Silva-Valencia J, Solari L. Protection of Prior SARS-CoV-2 Infection Against Different Variants, Including Omicron Descendants, in a Country with High Viral Transmission. *Pathogens and Immunity*. 2025;10(2):74–86. doi: 10.20411/pai.v10i2.760

Not health worker	536	(86.0)	2107	(87.6)	0.05	794	(83.5)	3101	(85.4)	0.05	2879	(83.7)	11123	(85.5)	0.05
Health worker	87	(14.0)	298	(12.4)		157	(16.5)	531	(14.6)		559	(16.3)	1888	(14.5)	
**Epidemiological week (2022)**															
17th week	1	(0.2)	0	(0.0)		0	(0.0)	0	(0.0)		0	(0.0)	0	(0.0)	
19th week	19	(3.0)	74	(3.1)		0	(0.0)	0	(0.0)		0	(0.0)	0	(0.0)	
20th week	16	(2.6)	64	(2.7)		3	(0.3)	12	(0.3)		0	(0.0)	0	(0.0)	
21st week	44	(7.1)	171	(7.1)		3	(0.3)	12	(0.3)		3	(0.1)	12	(0.1)	
22rd week	56	(9.0)	219	(9.1)		7	(0.7)	27	(0.7)		3	(0.1)	12	(0.1)	
23th week	81	(13.0)	316	(13.1)		15	(1.6)	57	(1.6)		20	(0.6)	76	(0.6)	
24th week	57	(9.1)	213	(8.9)		13	(1.4)	49	(1.3)		39	(1.1)	131	(1.0)	
25th week	75	(12.0)	279	(11.6)		34	(3.6)	134	(3.7)		103	(3.0)	400	(3.1)	
26th week	77	(12.4)	298	(12.4)		82	(8.6)	322	(8.9)		199	(5.8)	770	(5.9)	
27th week	75	(12.0)	292	(12.1)		130	(13.7)	515	(14.2)		272	(7.9)	1063	(8.2)	
28th week	55	(8.8)	212	(8.8)		164	(17.2)	642	(17.7)		531	(15.4)	2097	(16.1)	
29th week	49	(7.9)	195	(8.1)	0.06	150	(15.8)	582	(16.0)	0.04	691	(20.1)	2718	(20.9)	0.05
30th week	6	(1.0)	24	(1.0)		66	(6.9)	238	(6.6)		284	(8.3)	1078	(8.3)	
31st week	6	(1.0)	24	(1.0)		42	(4.4)	155	(4.3)		252	(7.3)	920	(7.1)	
32nd week	2	(0.3)	8	(0.3)		45	(4.7)	163	(4.5)		235	(6.8)	859	(6.6)	
33rd week	4	(0.6)	16	(0.7)		54	(5.7)	203	(5.6)		208	(6.1)	755	(5.8)	
34th week	0	(0.0)	0	(0.0)		49	(5.2)	180	(5.0)		205	(6.0)	753	(5.8)	
35th week	0	(0.0)	0	(0.0)		29	(3.0)	101	(2.8)		124	(3.6)	439	(3.4)	
36th week	0	(0.0)	0	(0.0)		32	(3.4)	120	(3.3)		122	(3.5)	428	(3.3)	
37th week	0	(0.0)	0	(0.0)		23	(2.4)	84	(2.3)		37	(1.1)	138	(1.1)	
38th week	0	(0.0)	0	(0.0)		4	(0.4)	16	(0.4)		43	(1.3)	154	(1.2)	
39th week	0	(0.0)	0	(0.0)		3	(0.3)	9	(0.2)		29	(0.8)	97	(0.7)	
40th week	0	(0.0)	0	(0.0)		2	(0.2)	7	(0.2)		15	(0.4)	47	(0.4)	
41st week	0	(0.0)	0	(0.0)		1	(0.1)	4	(0.1)		23	(0.7)	64	(0.5)	

Escobar-Agreda S, Araujo-Castillo RV, Pampa-Espinoza L, Silva-Valencia J, Solari L. Protection of Prior SARS-CoV-2 Infection Against Different Variants, Including Omicron Descendants, in a Country with High Viral Transmission. *Pathogens and Immunity*. 2025;10(2):74–86. doi: 10.20411/pai.v10i2.760

PCR, polymerase chain reaction; SMD, standardized mean difference.
*Cases and controls were exact matched one-to-four by sex, 10-year age group, macro-region, health worker status and epidemiological week of PCR test.
†SMD is the difference in the mean of a covariate between groups divided by the pooled standard deviation.

Table S7. Characteristics of matched cases (PCR-positive persons to Omicron BQ or XBB et al variants) and controls (PCR-negative persons) from the fifth study period.

	Omicron BQ et al					Omicron XBB et al				
	Cases (PCR-Positive)		Controls (PCR-Negative)		SMD†	Cases (PCR-Positive)		Controls (PCR-Negative)		SMD†
	Nro.	(%)	Nro.	(%)		Nro.	(%)	Nro.	(%)	
**Total**	753	(100)	2720	(100)		2036	(100)	7325	(100)	
**Sex**										
Female	441	(58.6)	1642	(60.4)	0.04	1219	(59.9)	4478	(61.1)	0.03
Male	312	(41.4)	1078	(39.6)		817	(40.1)	2847	(38.9)	
**Categorized age (10 years)**										
0-9 years	12	(1.6)	44	(1.6)	0.07	29	(1.4)	100	(1.4)	0.1
10-19 years	46	(6.1)	178	(6.5)		113	(5.6)	439	(6.0)	
20-29 years	121	(16.1)	456	(16.8)		398	(19.6)	1502	(20.5)	
30-39 years	158	(21.0)	594	(21.8)		453	(22.3)	1734	(23.7)	
40-49 years	148	(19.7)	524	(19.3)		329	(16.2)	1212	(16.5)	
50-59 years	117	(15.5)	435	(16.0)		338	(16.6)	1179	(16.1)	
60-69 years	103	(13.7)	350	(12.9)		226	(11.1)	756	(10.3)	
70-79 years	37	(4.9)	106	(3.9)		111	(5.5)	333	(4.5)	
80-89 years	10	(1.3)	31	(1.1)		35	(1.7)	69	(0.9)	
90-100 years	1	(0.1)	2	(0.1)		3	(0.1)	1	(0.0)	
**Macro-region**										
Lima-Callao	93	(12.4)	352	(12.9)	0.03	201	(9.9)	759	(10.4)	0.03
North	143	(19.0)	530	(19.5)		589	(28.9)	2185	(29.8)	
South	361	(47.9)	1300	(47.8)		256	(12.6)	921	(12.6)	
Center	108	(14.3)	363	(13.3)		290	(14.2)	971	(13.3)	

Escobar-Agreda S, Araujo-Castillo RV, Pampa-Espinoza L, Silva-Valencia J, Solari L. Protection of Prior SARS-CoV-2 Infection Against Different Variants, Including Omicron Descendants, in a Country with High Viral Transmission. *Pathogens and Immunity*. 2025;10(2):74–86. doi: 10.20411/pai.v10i2.760

PATHOGENS AND IMMUNITY
IN ASSOCIATION WITH CASE WESTERN RESERVE UNIVERSITY AND THE RICHARD J. FASENMYER FOUNDATION

West	48	(6.4)	175	(6.4)		700	(34.4)	2489	(34.0)	
**Health worker**										
Not health worker	549	(72.9)	2060	(75.7)	0.06	1613	(79.2)	5984	(81.7)	0.06
Health worker	204	(27.1)	660	(24.3)		423	(20.8)	1341	(18.3)	
**Epidemiological week (2022-2023)**										
43rd week	2	(0.3)	7	(0.3)		0	(0.0)	0	(0.0)	
45th week	20	(2.7)	62	(2.3)		18	(0.9)	57	(0.8)	
46th week	88	(11.7)	343	(12.6)		130	(6.4)	471	(6.4)	
47th week	92	(12.2)	336	(12.4)		284	(13.9)	1024	(14.0)	
48th week	150	(19.9)	551	(20.3)		409	(20.1)	1540	(21.0)	
49th week	140	(18.6)	509	(18.7)		321	(15.8)	1193	(16.3)	
50th week	98	(13.0)	351	(12.9)		384	(18.9)	1393	(19.0)	
51st week	75	(10.0)	258	(9.5)	0.07	230	(11.3)	803	(11.0)	0.07
52nd week	50	(6.6)	188	(6.9)		102	(5.0)	359	(4.9)	
1st week	14	(1.9)	47	(1.7)		73	(3.6)	241	(3.3)	
2nd week	12	(1.6)	34	(1.3)		36	(1.8)	120	(1.6)	
3rd week	6	(0.8)	17	(0.6)		18	(0.9)	45	(0.6)	
4th week	4	(0.5)	12	(0.4)		15	(0.7)	45	(0.6)	
5th week	1	(0.1)	1	(0.0)		11	(0.5)	20	(0.3)	
6th week	1	(0.1)	4	(0.1)		5	(0.2)	14	(0.2)	

PCR, polymerase chain reaction; SMD, standardized mean difference.
*Cases and controls were exact matched one-to-four by sex, 10-year age group, macro-region, health worker status and epidemiological week of PCR test.
†SMD is the difference in the mean of a covariate between groups divided by the pooled standard deviation.

Escobar-Agreda S, Araujo-Castillo RV, Pampa-Espinoza L, Silva-Valencia J, Solari L. Protection of Prior SARS-CoV-2 Infection Against Different Variants, Including Omicron Descendants, in a Country with High Viral Transmission. *Pathogens and Immunity*. 2025;10(2):74–86. doi: 10.20411/pai.v10i2.760

Table S8. Effectiveness of prior infection to prevent SARS-CoV-2 symptomatic reinfections by variants.

	Cases (PCR-Positive)		Controls (PCR-Negative)		Estimated effectiveness* (95% CI)
	Prior infection	Not prior infection	Prior infection	Not prior infection	
Lambda					
3-8 months	15	1,525	304	5,176	83.2 (72.8 to 89.6)
9-14 months	31	1,525	778	5,176	88.7 (83.7 to 92.2)
≥15 months	3	1,525	15	5,176	24.2 (-136.1 to 75.6)
Gamma					
3-8 months	9	676	154	2,275	81.4 (64.3 to 90.3)
9-14 months	33	676	332	2,275	67.7 (53.8 to 77.5)
≥15 months	2	676	26	2,275	88.7 (15.8 to 98.5)
Delta					
3-8 months	17	2,408	441	7,942	88.1 (80.5 to 92.7)
9-14 months	26	2,408	716	7,942	88.8 (83.3 to 92.5)
≥15 months	46	2,408	757	7,942	80.7 (73.8 to 85.8)
Omicron BA.1 et al					
3-8 months	24	1,726	171	6,256	49.3 (22.0 to 67.0)
9-14 months	113	1,726	590	6,256	32.4 (16.4 to 45.3)
≥15 months	185	1,726	988	6,256	33.8 (21.6 to 44.2)
Omicron BA.2.12.1					
3-8 months	15	536	364	1,627	88.1 (81.0 to 92.5)
9-14 months	14	536	71	1,627	28.1 (-18.2 to 56.3)
≥15 months	58	536	343	1,627	51.7 (36.5 to 63.3)
Omicron BA.4 et al					
3-8 months	64	751	529	2,455	65.3 (55.7 to 72.9)
9-14 months	17	751	114	2,455	59.3 (34.2 to 74.9)
≥15 months	119	751	534	2,455	30.3 (15.4 to 42.5)
Omicron BA.5 et al					
3-8 months	231	2,660	1,787	8,733	62.3 (56.9 to 67.0)
9-14 months	84	2,660	383	8,733	30.0 (12.5 to 43.9)
≥15 months	463	2,660	2,108	8,733	32.4 (25.0 to 39.1)
Omicron BQ et al					
3-8 months	15	462	260	1,493	85.5 (77.3 to 90.8)
9-14 months	148	462	505	1,493	8.1 (-10.8 to 23.7)
≥15 months	128	462	462	1,493	17.6 (0.0 to 32.2)
Omicron XBB et al					
3-8 months	67	1,248	556	4,430	65.0 (56.2 to 71.9)
9-14 months	314	1,248	1,068	4,430	-4.8 (-19.3 to 7.9)
≥15 months	407	1,248	1,271	4,430	-8.4 (-22.2 to 3.8)

*Effectiveness was estimated with a test-negative, nested case–control study design, matching in a 1:4 ratio according to sex, 10-year age group, region of origin, health workers, and calendar month of PCR testing from week 18 to 34 (2021) in patients with Lambda or Gamma variants; from week 35 to 51 (2021) in patients with Delta variant; from week 52 (2021) to 16 (2022) in patients with Omicron BA.1 et al variants; from week 17 to 41 (2022) in patients with Omicron BA.1, BA.2.12.1, BA.4 et al, or BA.5 et al variants; and from week 42 (2022) to 6 (2023) in patients with Omicron BQ or XBB et al variants.

Escobar-Agreda S, Araujo-Castillo RV, Pampa-Espinoza L, Silva-Valencia J, Solari L. Protection of Prior SARS-CoV-2 Infection Against Different Variants, Including Omicron Descendants, in a Country with High Viral Transmission. *Pathogens and Immunity*. 2025;10(2):74–86. doi: 10.20411/pai.v10i2.760

References

1. Ministerio de Salud del Perú. Coronavirus en el Perú: casos confirmados [Internet]. 2020 [citado el 23 de octubre de 2022]. Disponible en: https://www.gob.pe/8662-coronavirus-en-el-peru-casos-confirmados
2. Pampa-Espinoza L, Silva-Valencia J, Fernandez-Navarro M, Padilla-Rojas C, Solari L. Reinfections Are More Frequent Than Currently Considered in Countries With High Incidence of Coronavirus Disease 2019 (COVID-19) Cases Due to Stringent Definitions. Clin Infect Dis. el 15 de abril de 2022;74(8):1505–6.
3. Instituto Nacional de Salud. Instituto Nacional de Salud. 2022 [citado el 23 de mayo de 2022]. Secuenciación Genómica del virus SARS-CoV-2 en el Perú. Disponible en: https://web.ins.gob.pe/es/covid19/secuenciamiento-sars-cov2
4. Vargas-Herrera N, Araujo-Castillo RV, Mestanza O, Galarza M, Rojas-Serrano N, Solari-Zerpa L. SARS-CoV-2 Lambda and Gamma variants competition in Peru, a country with high seroprevalence. Lancet Reg Health – Am [Internet]. el 1 de febrero de 2022 [citado el 23 de octubre de 2022];6. Disponible en: https://www.thelancet.com/journals/lanam/article/PIIS2667-193X(21)00108-3/fulltext

Escobar-Agreda S, Araujo-Castillo RV, Pampa-Espinoza L, Silva-Valencia J, Solari L. Protection of Prior SARS-CoV-2 Infection Against Different Variants, Including Omicron Descendants, in a Country with High Viral Transmission. *Pathogens and Immunity*. 2025;10(2):74–86. doi: 10.20411/pai.v10i2.760
